# Neutrophil Extracellular Trap Scores Predict 90-Day Mortality in Hepatitis B-Related Acute-on-Chronic Liver Failure

**DOI:** 10.3390/biomedicines12092048

**Published:** 2024-09-09

**Authors:** Yi Zhang, Ke Shi, Bingbing Zhu, Ying Feng, Yao Liu, Xianbo Wang

**Affiliations:** Center of Integrative Medicine, Beijing Ditan Hospital, Capital Medical University, Beijing 100054, China; dejavu_zy@163.com (Y.Z.); 13253691368@163.com (K.S.); zhubb1220@163.com (B.Z.); ann_fengying@163.com (Y.F.); liuyao.ly@163.com (Y.L.)

**Keywords:** neutrophil extracellular traps, nomogram, prognostic model, inflammatory response, hepatitis B-related acute-on-chronic liver failure

## Abstract

Hepatitis B virus (HBV)-related acute-on-chronic liver failure (ACLF) is associated with pronounced systemic inflammation, and neutrophil extracellular traps (NETs) are key components of this response. The primary objective of this study was to establish an NET-related scoring system for patients with HBV-ACLF. A prospective training cohort of 81 patients from the Beijing Ditan Hospital was included. The concentrations of NET markers (cell-free DNA, myeloperoxidase DNA [MPO-DNA], and citrullinated histone H3) in peripheral blood were quantified. Random survival forest, LASSO regression, and multivariate Cox regression analyses were used to identify prognostic factors associated with 90-day mortality in ACLF patients and develop a nomogram for visualization, which was followed by evaluation in a validation cohort (*n* = 40). NET-related marker levels were significantly higher in the non-survival group than in the survival group (*p* < 0.05). The NET score was constructed by combining MPO-DNA, neutrophil-to-lymphocyte ratio, and age data. The score’s diagnostic effectiveness, assessed by the area under the curve, yielded values of 0.83 and 0.77 in the training and validation sets, respectively, markedly surpassing those of other established models (*p* < 0.05). In both groups, the 90-day mortality rates were 88.8% and 75.0%, respectively, for patients categorized as high risk and 18.0% and 12.5%, respectively, for those classified as low risk.

## 1. Introduction

Acute-on-chronic liver failure (ACLF) is defined as acute liver failure in Asian patients with an underlying chronic liver disease or cirrhosis [1] and is characterized by a rapid increase in liver function and a rapid decline in coagulation function [2]. In developing countries, 70% of cases are caused by hepatitis B virus (HBV) infection [3], and 30–70% of patients die within 90 days due to rapid disease progression. Currently, the only effective treatments for patients with ACLF are intensive care support and emergency liver transplantation. Consequently, there is a critical need to promptly identify patients with poor prognoses. This necessitates the use of a highly accurate prognostic tool that can facilitate timely and appropriate clinical interventions. Several models have been used to predict ACLF prognosis, including the Model for End-stage Liver Disease (MELD) [4], MELD-Na score [5], Chronic Liver Failure Consortium ACLF (CLIF-C ACLF) [6], and Chinese Group on the Study of Severe Hepatitis B ACLF (COSSH-ACLF) [7]. However, their detection indicators primarily involve routine biochemical values and do not target the underlying pathogenesis of HBV-ACLF. Moreover, the inclusion criteria for these indicators are complex and inconvenient for calculation. Consequently, these scores are often used as advanced liver transplantation scores for patients who may miss out on the best possible treatment. Thus, given that a patient’s mortality risk can be affected in HBV-ACLF by inflammatory responses [8], the accuracy of these conventional models in predicting the prognosis of ACLF may be limited.

Over the past decade, significant progress has been made in the study of the pathogenesis of ACLF. Neutrophils, which are crucial components of the circulating innate immune cell population, are considered the predominant immune cell type infiltrating the liver during liver injury [9]. Its main functions include phagocytosis, the release of reactive oxygen species (ROS), the formation of neutrophil extracellular traps (NETs), the release of cytokines, and other pro-inflammatory functions [10]. NETs trigger widespread and intense inflammatory responses within the body. Recent studies have acknowledged their roles as key drivers in the pathogenesis of a diverse array of diseases, including tumors, sepsis, and cardiovascular diseases [11,12]. They are also found in the plasma of patients with acute liver failure (ALF). Furthermore, elevated plasma NET levels are correlated with adverse outcomes in ALF cases [13]. However, there have been few reports on NET-related markers in patients with HBV-ACLF.

Therefore, we aimed to establish a nomogram with NET markers to predict 90-day mortality in patients with HBV-ACLF, which could help clinicians in the early surveillance of high-risk patients and highlight the potential for targeting NETs to mitigate adverse outcomes.

## 2. Materials and Methods

### 2.1. Patient Enrollment

**Figure 1:**
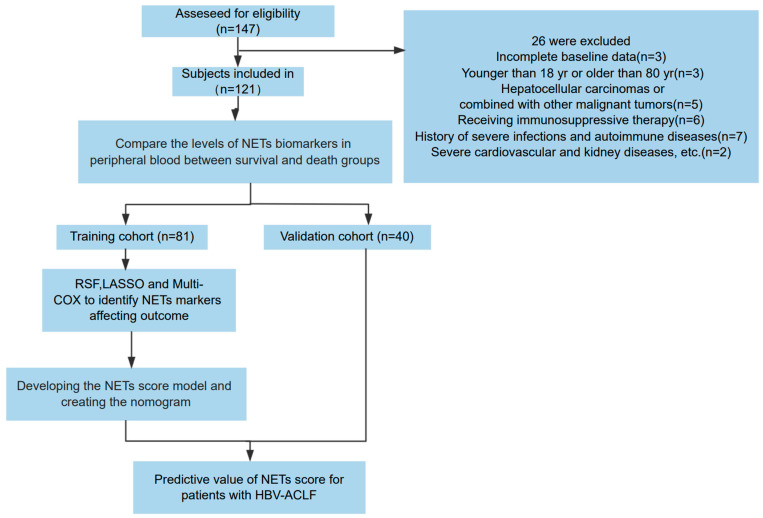
Flow chart of the clinical research. A total of 121 patients with hepatitis B virus-related acute-on-chronic liver failure (HBV-ACLF) underwent a screening process to determine their eligibility for inclusion in the research. Following this screening, 81 participants were allocated to the training group, while 40 were designated to the validation group.

In the training cohort, we prospectively included 81 patients with HBV-ACLF admitted to the Beijing Ditan Hospital Affiliated to Capital Medical University between January 2018 and January 2022. Exclusions included incomplete baseline data, age younger than 18 years or older than 80 years, hepatocellular carcinomas or combined malignant tumors, receiving immunosuppressive therapy, history of severe infections or autoimmune diseases, severe cardiovascular or kidney diseases, or other severe conditions. Using the same inclusion and exclusion criteria, we enrolled a validation cohort of 40 patients between February 2022 and October 2023. The study process is illustrated in Figure 1. All patients were chronic HBV carriers and met the diagnostic criteria of the Asia-Pacific Association for the Study of the Liver (APASL) for ACLF [14]. Comparative analysis indicated no significant differences in various parameters between the training and validation cohorts (Table 1).

Clinical data and laboratory variables were collected from electronic medical records at enrollment. All patients received standardized medical treatment during hospitalization. This study was approved by the Ethics Committee of Beijing Ditan Hospital (Beijing, China), and all participants provided written informed consent. The study adhered to the ethical principles of the Declaration of Helsinki.

### 2.2. Blood Sample Collection and Storage Process

All blood samples were collected within 48 h of patient admission. We collected 6 mL of patient blood in EDTA tubes (BD, CAT 367863) and centrifuged them at 2000 rpm for 10 min at 4 °C. The supernatant plasma samples were then separated and stored in a −80 °C freezer. All patient samples were processed in the same manner before undergoing unified testing after the completion of sample separation.

### 2.3. NET Measurement

In this study, we quantified the components of NETs, including cell-free DNA (cfDNA), myeloperoxidase DNA (MPO-DNA) complexes, and citrullinated histone H3 (CitH3).

#### 2.3.1. cfDNA Detection

cfDNA was detected using the PicoGreen kit (Thermo Fisher Scientific, Waltham, MA, USA). First, 20× concentrated PE was diluted to a 1× concentration with ddH_2_O, and the 1× PE was used to obtain a standard gradient. Then, 50 µL of TE-diluted standards and plasma samples were added to each well of a 96-well plate and incubated at room temperature on a shaker at 320 rpm for 2 h. Finally, 50 µL of diluted PicoGreen was added to each well, and the plate was incubated in the dark at room temperature for 2 min. Fluorescence intensity was measured using a microplate reader with an excitation wavelength of 480 nm and an emission wavelength of 520 nm.

#### 2.3.2. MPO-DNA Detection

First, 5 µg/mL of an MPO antibody (Bio-Rad, Hercules, CA, USA) diluted in coating buffer was added to each well of a 96-well plate, and the plate was incubated overnight at 4 °C. The following day, the wells were washed three times with washing buffer, and nonspecific binding sites were blocked with a coating buffer containing 1% BSA for 90 min. After washing, 60 µL of plasma samples and 3 µL of an anti-DNA-POD antibody (Cell Death Detection ELISAPLUS, Roche, Basel, Switzerland) were added to each well and incubated for 2 h at room temperature on a shaker. Finally, 100 µL of diluted ABTS substrate was added to each well, incubated in the dark for 40 min, and absorbance was measured at 405 nm.

#### 2.3.3. CitH3 Detection

The generation of CitH3 was detected using the Citrullinated Histone H3 (Clone 11D3) ELISA kit (Cayman, Ann Arbor, MI, USA). First, 100 µL of standards or plasma samples were added to each well of an Anti-Citrullinated Histone H3 ELISA Strip Plate and incubated for 2 h at room temperature on an orbital shaker. The wells were then emptied and washed five times with washing buffer, and 100 µL of the HRP conjugate working solution was added to each well. The plate was incubated for 1 h at room temperature. Finally, 100 µL of the TMB substrate solution was added to each well, the plate was incubated in the dark at room temperature for 30 min, and absorbance was measured at 450 nm.

### 2.4. Statistical Analysis

Variables that adhered to a normal distribution are expressed as means and standard deviations (SDs). Conversely, for variables exhibiting a non-normal or skewed distribution, data are presented as median and interquartile range (IQR). Continuous variables were compared between the two groups using the *t*-test or Mann–Whitney U test, while categorical variables were compared using the chi-square test. To visualize the potentially nonlinear relationship between NET components in the blood and 90-day mortality in patients, a restricted cubic spline (RCS) model with three knots was used. Risk factors were assessed using the random survival forest (RSF) and LASSO regression. A multivariate Cox proportional risk model was used, and the final variables were determined using a stepwise selection method. The concordance index (C-index) and receiver operating characteristic (ROC) curve, represented by the area under the curve (AUC), were used to evaluate the performance of the models. Additionally, the “rms” R package was utilized to generate the graphical representation of the multivariate analysis results in the form of a modal graph.

We compared the novel NET score with the MELD-Na, CLIF C-ACLF, and COSSH-ACLF scores using the Delong test [15]. A calibration plot was constructed to evaluate the agreement between the probability of survival and the observed probability. In addition, decision curve analysis (DCA) was performed to assess the clinical net benefits using the “ggDCA” R package. Survival probability was determined using Kaplan–Meier curves, and differences were compared using the log-rank test. Statistical analyses were performed using the R software (version 4.1.2; R Foundation, Vienna, Austria). Statistical significance was set at *p* < 0.05.

## 3. Results

### 3.1. Baseline Characteristics of Patients with HBV-ACLF

Thirty-four patients (41.9%) in the training cohort and 15 patients (37.5%) in the validation cohort died within 90 days. The baseline characteristics of patients at admission are summarized in Table 1. The median cfDNA, MPO-DNA, and CitH3 levels were 242.67, 0.24, and 3.03 in the training cohort, respectively; these values were 246.43, 0.26, and 2.84 in the validation cohort, respectively. There was no significant difference between the baseline data of the two cohorts (all *p* > 0.05).

### 3.2. Expression Levels of NET-Related Markers in HBV-ACLF Patients

**Figure 2:**
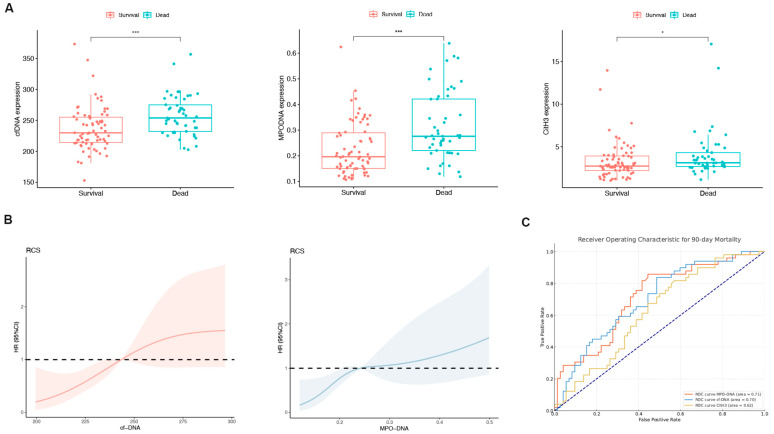
The levels of neutrophil extracellular trap (NET) biomarkers exhibited significant differences between patients in the survival and non-survival groups. (**A**) Differences in the peripheral blood NET markers (cell-free DNA [cfDNA], myeloperoxidase DNA complexes [MPO-DNA], and citrullinated histone H3 [CitH3]) between the survival and non-survival groups of HBV-ACLF patients. * *p* < 0.05, *** *p* < 0.001. (**B**) Associations among cfDNA, MPO-DNA, and 90-day mortality according to a restricted cubic spline regression model (both *p*-values for linearity: >0.05). (**C**) Receiver operating characteristic (ROC) curve for single-factor NET markers predicting 90-day mortality in HBV-ACLF patients.

Patients were divided into a survival group (*n* = 64) and a non-survival group (*n* = 57), and NET marker levels in the peripheral blood were compared between the groups. With the exception of CitH3, the MPO-DNA and cfDNA levels exhibited a significant increase in the non-survival group (both *p* < 0.05; Figure 2A). The RCS plot indicated a relatively low risk within the lower range of cfDNA and MPO-DNA, which increased thereafter, showing that cfDNA and MPO-DNA were positively and linearly correlated with the 90-day mortality (*p* > 0.05; Figure 2B). In the assessment of individual prognostic factors for 90-day mortality, the AUC for MPO-DNA was determined to be 0.71 with a 95% confidence interval (CI) of 0.62–0.80. Similarly, for cfDNA, the AUC was 0.70 (95% CI: 0.60–0.79), and for CitH3, it was 0.62 (95% CI: 0.52–0.72), as depicted in Figure 2C. These results indicate that peripheral blood NET markers were elevated in the deceased group compared to the survival group.

### 3.3. Prognostic Model Based on LASSO-Cox Regression

**Figure 3:**
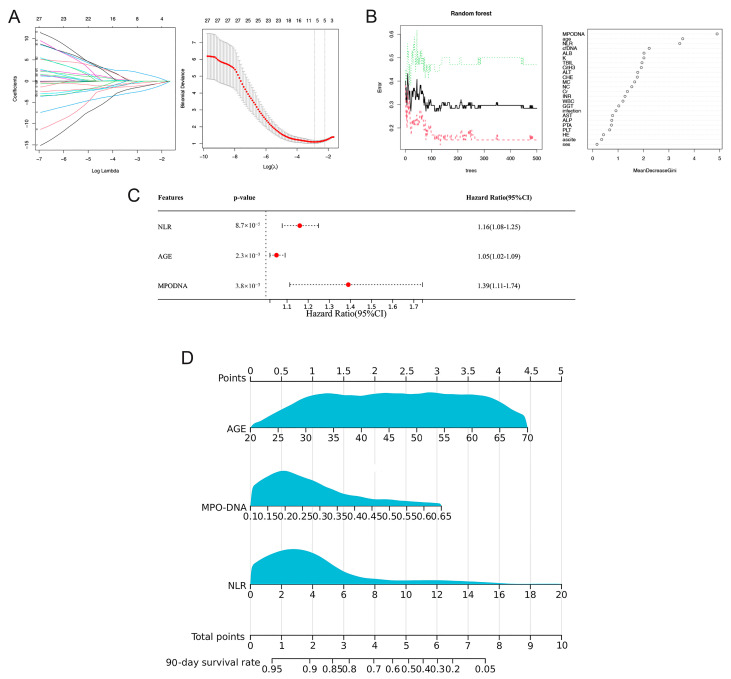
Selection of prognostic factors for 90-day mortality in HBV-ACLF patients and construction of the prognostic model and visual nomogram. (**A**) The LASSO coefficient profiles of the clinical features and the selection of the optimum parameter (lambda) in the LASSO model were determined using ten-fold cross-validation. (**B**) Predictors based on random survival forest (RSF) analysis, including the error rate of the RSF and out-of-bag variable importance ranking. (**C**) Multivariate Cox regression to predict recurrence based on RSF analysis and LASSO regression. (**D**) Visual nomogram of the NET score predicting the 90-day mortality of patients with HBV-ACLF.

In the training cohort, LASSO regression analysis was used to select potential prognostic risk factors. The optimum parameter (lambda) selection in the LASSO model was performed using ten-fold cross-validation employing the minimum criteria. The variation characteristics of the coefficients of these variables are shown in Figure 3A. Nine prognostic indicators were screened, including age, sex, MPO-DNA, cfDNA, sodium (Na), creatinine (Cr), alanine transaminase (ALT), hepatic encephalopathy (HE), and neutrophil-to-lymphocyte ratio (NLR). Multivariate Cox regression analysis showed that NLR (HR = 1.16, 95% CI: 1.08–1.25; *p* < 0.01), MPO-DNA (HR = 1.39, 95% CI: 1.11–1.74; *p* < 0.01), and age (HR = 1.05, 95% CI: 1.02–1.09; *p* < 0.01) were independent risk factors for 90-day mortality (Figure 3B).

### 3.4. Prognosis Model Based on RSF-Cox Analysis

To obtain a more precise predictive model, we employed the RSF method. This approach began with a random sampling of data from the modeling group to establish the foundational structure of the model. When the number of trees (ntree) was 400, the error rate of the model was the lowest. After adjusting the parameters to obtain the best performance model, we used the variable importance (VIMP) and minimum depth methods to comprehensively rank the importance of the input variables.

Analysis of the variable importance scores identified six key variables with scores exceeding 0.2. These included cfDNA, age, MPO-DNA, albumin (Alb), NLR, and potassium (K) (Figure 3C). Subsequently, these six factors were incorporated into a multi-Cox regression analysis. This analysis corroborated the findings of the LASSO-Cox model, reiterating the significance of the three primary factors: MPO-DNA, age, and NLR.

### 3.5. Discrimination and Calibration of the Nomogram

**Figure 4:**
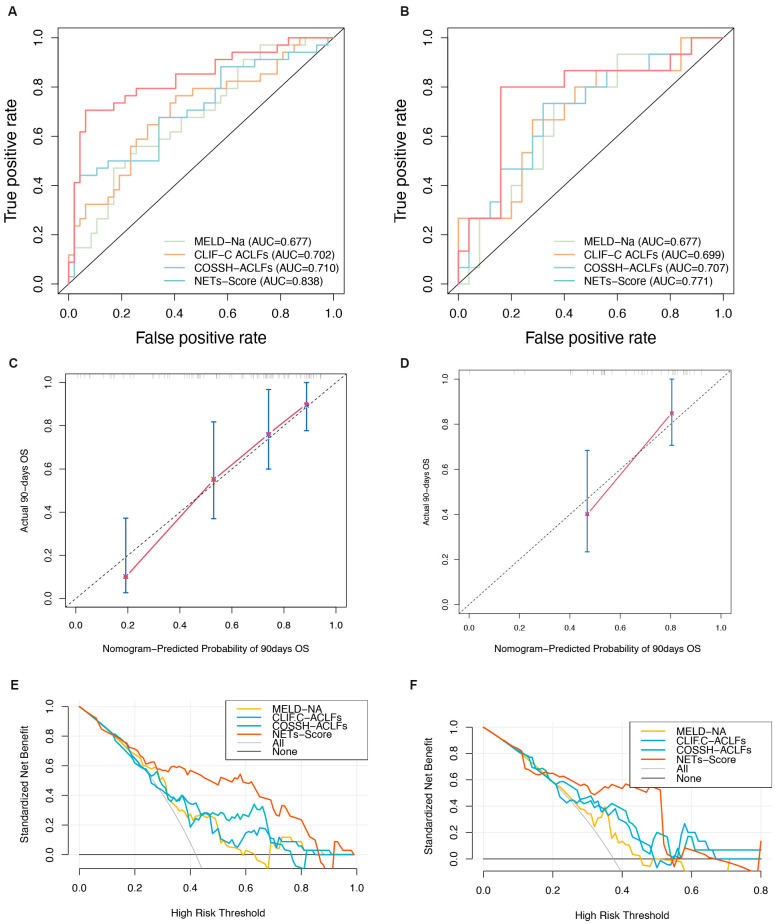
Evaluation of the predictive performance of the NET score. (**A**,**B**) Receiver operating characteristic curves of the NET score and other models. (**C**,**D**) Calibration plots of the NET score and other models. (**E**,**F**) Decision curves of the NET score and other models.

Screening using different statistical methods identified MPO-DNA, age, and NLR as independent risk factors for the 90-day mortality. Therefore, we combined these three indicators to establish the NET score. The 90-day survival probability formula for HBV-ACLF patients is NET score = 0.053 × AGE + 2.976 × MPO-DNA + 0.148 × NLR. To make the scoring system more convenient to use, a visual nomogram was constructed (Figure 3D). The results showed that compared to traditional models, the NET score demonstrates superior predictive performance and outperforms the other three models in terms of AUC values. In the training and validation cohorts, the AUC values for the NET score were 0.83 (95% CI: 0.74–0.93) and 0.77 (95% CI: 0.94–0.60), respectively, surpassing those of the MELD-Na score (0.68 [95% CI: 0.56–0.79] and 0.68 [95% CI: 0.51–0.85], respectively), CLIF-C ACLF (0.70 [95% CI: 0.62–0.82] and 0.66 [95% CI: 0.61–0.84], respectively), and COSSH-ACLF (0.71 [95% CI: 0.61–0.83] and 0.72 [95% CI: 0.62–0.89], respectively) (all *p* < 0.05, Figure 4A,B). Furthermore, the c-index values of the NET score in the two cohorts were 0.76 and 0.71, respectively. Additionally, the calibration curve showed that the NET score was in good agreement with the observed probability in the training and validation cohorts (Figure 4C,D), and DCA curves showed that the NET score had significant net clinical benefits when compared to the MELD-Na, CLIF-C ACLF, and COSSH-ACLF (Figure 4E,F). In both the training group and the validation group, the sensitivity, specificity, positive predictive value, and negative predictive value of the NET score were significantly higher than those of other classical models (Table 2).

### 3.6. Risk Stratification for Patients with HBV-ACLF

**Figure 5:**
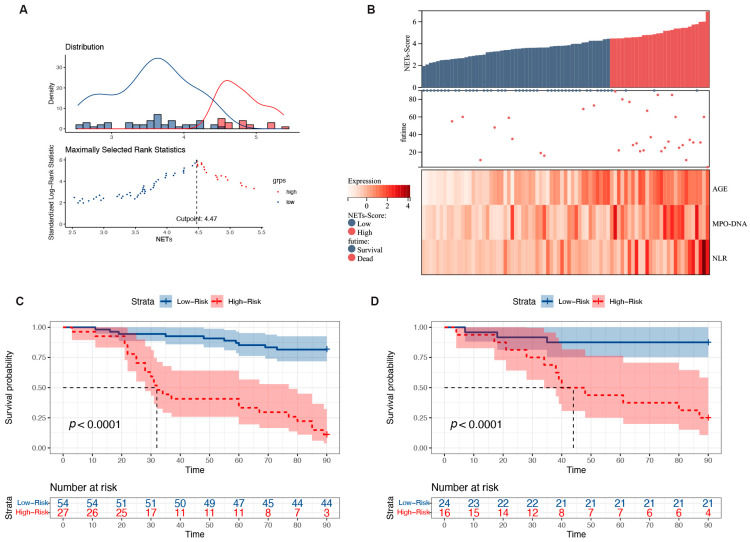
Relationship between different expression levels of the predictors included in the NET score and 90-day mortality in patients with HBV-ACLF. (**A**) The optimal best cutoff value was calculated according to the nomogram. (**B**) Associations among the different risk scores, patient follow-up times, outcome events, and changes in the expression of the three predictors included in the NET score. (**C**) Survival curves of mortality in the training group based on the best cutoff value of the nomogram using the Kaplan–Meier method. (**D**) Validation of the survival rates.

Based on the NET score, the HBV-ACLF patient cohort was subjected to risk scoring. The optimal cutoff value was 4.5, and patients were divided into high- and low-risk groups. The prognostic model was visualized using the risk score and three risk factors (Figure 5A). A higher number of deaths occurred predominantly on the right side, indicating a high-risk score. MPO-DNA expression, age, and NLR were all significant risk factors that increased as the patients’ risk scores increased (Figure 5B). The 90-day mortality rates in the training and validation cohorts were 18.0% and 12.5% in the low-risk group (<4.5), respectively, whereas these were 88.8% and 75.0% in the high-risk group (≥4.5), respectively (both *p* < 0.0001; Figure 5C,D). Additionally, the hazard ratio (HR) for high-risk versus low-risk patients was 9.07 (4.27–19.27) in the training group and 8.61 (2.40–18.84) in the validation group. According to the cutoff value, the low cutoff value of the NET score achieved a negative predictive value of 93.6% in the training cohort and 88.3% in the validation cohort for excluding patients with a good 90-day prognosis (Table 2).

## 4. Discussion

Patients with HBV-ACLF have exceptionally high mortality rates. Consequently, the early identification of high-risk patients is of paramount importance. The existing prognostic models such as the MELD-Na, CLIF-C ACLF, and COSSH-ACLF scores have been utilized for this purpose. However, it is important to note that the MELD-Na was originally developed for patients with cirrhosis rather than those with ACLF. This distinction in the pathogenesis between the two conditions may have limited the predictive accuracy of the MELD-Na score in our study cohort [16]. Additionally, the CLIF-C ACLF model, which was specifically designed to assess the mortality of patients with ACLF in Europe, performs poorly in cases of ACLF caused by viral infections. Conversely, the COSSH-ACLF model, a scoring system established jointly by 13 centers in China [7] to predict the mortality of patients with HBV-ACLF, demonstrated good predictive ability in this study. However, this calculation is complex and requires various clinical and laboratory parameters. In conclusion, because of the complexity of the disease etiologies and the involvement of multiple organs in later stages, accurately predicting individual patient outcomes in those with HBV-ACLF remains a challenging task.

Recently, extensive research on the pathogenesis of ACLF has led to the identification of several novel biomarkers. Significant characteristics of HBV-ACLF include a systemic inflammatory response, heightened decompensation of the liver, and multi-organ failure. Contemporary research has revealed that throughout the progression of ACLF, there is a substantial infiltration of neutrophils into the liver. These neutrophils, identified as the primary effector cells during this phase of the disease, participate in the progression of ACLF and potentially serve as therapeutic targets [17,18]. Consequently, we hypothesized that the abundant release of NETs is a contributing factor to the poor prognosis of patients with HBV-ACLF. A study published in Hepatology in 2022 [13] indicates that NETs markers are elevated in patients with acute liver failure and are associated with mortality. Furthermore, elevated levels of NETs are also observed in the peripheral blood and liver tissues of both patients and mouse models with fatty liver disease [12] and liver cancer [19]. At the same time, alcohol impairs the ability of macrophages to clear NETs from the liver, leading to an accumulation of excessive NETs, which exacerbates the inflammatory response in the liver in alcoholic hepatitis. Additionally, NETs can promote the formation of microvascular thrombosis in the hepatic sinusoids, increasing portal pressure [20]. Two studies on ACLF have also confirmed that neutrophils exhibit significantly impaired phagocytic and oxidative burst capacities, while their ability to form NETs is increased, especially in patients with poor prognosis [21,22]. Furthermore, due to the potent pro-inflammatory properties of NETs, their role in other inflammation-related diseases, such as inflammatory lung disease [23], sepsis [24], and COVID-19 [25], is receiving increasing attention. These findings suggest that NETs play a significant role in various liver diseases, including liver failure, and are associated with disease progression. The mechanisms through which NETs may lead to mortality are as follows: (1) During liver failure, a large number of damaged hepatocytes release damage-associated molecular patterns, which promote neutrophil activation and NET release, resulting in a fatal inflammatory cycle. (2) NETs can promote thrombosis by adhering to platelets, exacerbating the patient’s bleeding tendency. (3) Infiltrating neutrophils, upon reaching the site of inflammatory injury, exacerbate the existing damage by promoting the release of free radicals and proteolytic enzymes. Through these pathways, NETs contribute to irreversible liver damage, systemic coagulation dysfunction, and potentially trigger a systemic inflammatory response, leading to septic shock and accelerating the patient’s death. Initially, by quantifying the NET-related biomarkers, namely cfDNA and MPO-DNA, in the plasma of patients, we observed a significant increase in these markers among those who died. Specifically, the RCS curve indicated that when MPO-DNA exceeds 0.24 and cfDNA surpasses 240, the risk of death increases progressively with higher values. However, CitH3, another NET marker, was not an independent predictor of death in patients with HBV-ACLF in our study. Shedding light on this aspect, recent studies have shown that CitH3 is not essential for NET formation [26]; instead, the PAD4 protein can mediate NET formation independent of CitH3. This suggests that NET production mediated by the PAD4 pathway may contribute to the death of patients with HBV-ACLF. Moreover, targeting NETs has already been partially applied in disease treatment. For instance, in patients with cystic fibrosis [27] and autoimmune lupus nephritis [28], the injection or inhalation of low doses of DNase I (a specific inhibitor that breaks down NETs) has shown promising therapeutic effects. Recently, a novel PAD4 inhibitor, ZD-E-1M, has been developed to suppress NET formation in humans and mice, which can significantly reduce chemotherapy-induced pain and inhibit the neurotoxicity of tumors [29]. While the efficacy of NET inhibitors in patients with various diseases, particularly those with liver failure, requires further investigation, recent basic research has indicated that the degradation of NETs using DNase I, the inhibition of key proteins involved in NET formation, or the knockout of critical genes responsible for NET production can all suppress NET formation and improve liver failure in mouse models [30,31]. These suggest that NET inhibitors may hold significant potential for application in patients with liver failure.

Multivariate regression analysis revealed that NLR, MPO-DNA, and the quantity and function of neutrophils are independent predictors of 90-day mortality, providing insights into both the patient’s liver damage and overall inflammatory condition. Additionally, we found that increasing age was related to patient mortality, which may indicate a stronger pro-inflammatory effect in the neutrophils of older patients. Therefore, we combined NLR, MPO-DNA, and age to establish the NET score and evaluated its predictive capability. The AUCs of the NET score were 0.83 and 0.77 in the training and validation cohorts, respectively. Notably, patients scoring <4.5 demonstrated a 90-day mortality rate of 18.0%, indicative of a generally favorable prognosis, allowing them to circumvent more intricate and costly tests. Conversely, when a patient’s baseline score was >4.5, the mortality rate reached 88.8%, suggesting the need for closer monitoring and potentially necessitating early ICU admission or arrangements for liver transplantation.

Recently, the release of NETs in various diseases has received considerable research attention. For instance, in two large-sample cohorts, cfDNA levels were associated with the prognosis of patients with acute liver failure [13] and sepsis [32]. Although cfDNA is considered a marker of NETs, it may also originate from other necrotic and apoptotic cells [33]. In patients with ACLF, a rapid and massive necrosis of hepatocytes is observed. Due to severe liver cell damage and the rapidly progressing inflammatory response, the function of Kupffer cells may be suppressed, leading to the failure of timely clearance of apoptotic hepatocytes. At this point, DNA fragments exposed after the rupture of apoptotic bodies or uncleared apoptotic bodies may pass through tissue gaps and enter the blood or lymphatic system, entering systemic circulation [34,35]. Therefore, quantification based solely on cfDNA cannot represent the level of NETs in the body, and it is necessary to combine cfDNA with other markers. Notably, some studies suggest that MPO-DNA in the peripheral blood circulation is more stable than cfDNA and difficult to degrade using NET inhibitors such as DNase I [36]. Therefore, the role of MPO-DNA in disease diagnosis, treatment, and prognosis deserves attention. On the other hand, CitH3, originating from the guanidination of arginine residues in histone H3, is also a product of NETs. To address this, further studies are required to elucidate whether CitH3 alone can adequately represent NET formation or if it needs to be combined with other indicators for accurate quantification.

Based on substantial neutrophil release and systemic inflammation, we developed a highly accurate NET score to predict the 90-day mortality rate of patients with HBV-ACLF. However, a major limitation of this study is that although we had both a training cohort and a validation cohort, the sample size of both cohorts was small, and the data were from the same hospital; therefore, the evidence was slightly insufficient. In addition, a subgroup analysis was not possible because of the small patient cohort. Therefore, to enhance the robustness and generalizability of our findings, we plan to include more research centers and a larger patient sample in future studies to further validate and extend our results. It is also essential to consider grouping patients based on potential etiologies and conducting more targeted research on patients with different underlying causes. Although there are currently no reports of routine diseases or medications affecting NET formation and their biomarker quantification in vivo, future studies should include more detailed stratified analyses to clarify the specific impact of various comorbidities and medication histories on NET levels. This will further enhance the rigor and clinical relevance of the research. Finally, future studies should involve longer-term dynamic monitoring of patients to better understand the relationship between changes in NET biomarker levels and disease progression.

## 5. Conclusions

NETs are emerging as potentially predictive biomarkers of 90-day mortality in patients with HBV-ACLF. Models that include NETs, which reflect neutrophil activation and extensive inflammatory responses in the body, are better predictors of short-term mortality than other models. According to this NET-related scoring system, the 90-day mortality rate of high-risk patients can reach 88.8% and will assist clinicians in early patient stratification and management, allowing the formulation of therapeutic strategies to improve patient outcomes.

## Figures and Tables

**Table 1:** Demographics and clinical characteristics of the training and validation cohorts.

	Training Cohort (*n* = 81)	Validation Cohort (*n* = 40)	*p* Values
Patients background			
Age, yr	46 (36, 58)	47.5 (38.25, 54)	0.732
Gender (male/female)	63 (18)	36 (4)	0.165
Ascite, *n* (%)	68 (84)	36 (90)	0.533
Abdominal infection	37 (46)	16 (40)	0.691
Hepatic Encephalopathy, *n* (%)	36 (44)	15 (38)	0.595
NETs–related indicators			
MPO-DNA	0.24 (0.16, 0.33)	0.26(0.17, 0.34)	0.862
cf-DNA	242.67 (219, 268.86)	246.43 (220.84, 260.84)	0.985
CitH3	3.03 (2.47, 3.94)	2.84 (2.51, 3.93)	0.217
Laboratory parameters			
ALT (U/L)	334.3 (103, 695.9)	252.1 (113.58, 541.85)	0.932
AST (U/L)	212.4 (103, 391.6)	268.65 (146.52, 474.12)	0.283
TBil (µmol/L)	244.6 (177.1, 333.40)	281.75 (219.4, 349.38)	0.106
γ-GGT (U/L)	82.3 (46, 112.6)	96.4 (57.08, 142.98)	0.136
ALB (g/L)	31.1 (28.6, 33.3)	32.05 (29.8, 35.35)	0.069
Cr (µmol/L)	72 (60, 81.7)	67.2(58.45, 80.55)	0.512
INR	2.22 (1.9, 2.75)	2.15 (2, 2.41)	0.381
PTA (%)	35 (27, 39)	36 (30.8, 40)	0.178
WBC (10^9^/L)	6.14 (4.4, 8.3)	5.16 (4.46, 6.69)	0.279
NLR	2.74 (2.07, 4.78)	3.58 (2.25, 5.29)	0.365
K^+^ (mmol/L)	3.74 (3.52, 4.1)	3.99 (3.63, 4.24)	0.026
NA^+^ (mmol/L)	138.5 (135.4, 142.4)	137.5 (132.75, 139.85)	0.081

Categorical values are shown as *n* (%). Continuous variables are shown as median (interquartile range, IQR). Abbreviations: NETs, neutrophil extracellular traps; MPO-DNA, myeloperoxidase DNA complexes; cf-DNA, cell-free DNA; CitH3, citrullinated histone H3; ALT, alanine aminotransferase; AST, aspartate aminotransferase; TBil, total bilirubin; γ-GGT, gamma-glutamyl transferase; ALB, albumin; ALP, alkaline phosphatase; CHE, cholinesterase; Cr, creatinine; INR, international normalized ratio; PTA, prothrombin activity; PLT, platelet count; WBC, white blood cell; NC, neutrophil count; LC, lymphocyte count; MC, monocyte count; K^+^, kalemia; NA^+^, natremia; NLR, neutrophil–lymphocyte ratio.

**Table 2:** Predictive performance of the NET score in the training and validation cohorts.

Cutoff Value	Sensitivity (%) (95% CI)	Specificity (%) (95% CI)	PPV (%) (95% CI)
Training cohort (*n* = 81)			
NET score	87.5%	77.2%	71.8%
MELD-Na	61.5%	67.3%	47.1%
CLIF-C ACLF	65.5%	71.2%	55.9%
COSSH-ACLF	56.6%	85.7%	51.1%
Validation cohort (*n* = 40)			
NET score	70.6%	86.9%	80%
MELD-Na	52.3%	78.9%	73.33%
CLIF-C ACLF	50%	85.7%	48%
COSSH-ACLF	55%	80%	64%

## Data Availability

The original contributions presented in the study are included in the article. Further inquiries can be directed to the corresponding author.

## References

[1] Sarin S.K., Kedarisetty C.K., Abbas Z., Amarapurkar D., Bihari C., Chan A.C., Chawla Y.K., Dokmeci A.K., Garg H., Ghazinyan H. (2014). Acute-on-chronic liver failure: Consensus recommendations of the Asian Pacific Association for the Study of the Liver (APASL) 2014. Hepatol. Int..

[2] Arroyo V., Moreau R., Kamath P.S., Jalan R., Ginès P., Nevens F., Fernández J., To U., García-Tsao G., Schnabl B. (2016). Acute-on-chronic liver failure in cirrhosis. Nat. Rev. Dis. Primers.

[3] Zhang Q., Li Y., Han T., Nie C., Cai J., Liu H., Liu Y. (2015). Comparison of current diagnostic criteria for acute-on-chronic liver failure. PLoS ONE.

[4] Malinchoc M., Kamath P.S., Gordon F.D., Peine C.J., Rank J., ter Borg P.C. (2000). A model to predict poor survival in patients undergoing transjugular intrahepatic portosystemic shunts. Hepatology.

[5] Hernaez R., Liu Y., Kramer J.R., Rana A., El-Serag H.B., Kanwal F. (2020). Model for end-stage liver disease-sodium underestimates 90-day mortality risk in patients with acute-on-chronic liver failure. J. Hepatol..

[6] Hernaez R., Solà E., Moreau R., Ginès P. (2017). Acute-on-chronic liver failure: An update. Gut.

[7] Wu T., Li J., Shao L., Xin J., Jiang L., Zhou Q., Shi D., Jiang J., Sun S., Jin L. (2018). Development of diagnostic criteria and a prognostic score for hepatitis B virus-related acute-on-chronic liver failure. Gut.

[8] European Association for the Study of the Liver (2023). EASL Clinical Practice Guidelines on Acute-on-Chronic Liver Failure. J. Hepatol..

[9] Kolaczkowska E., Kubes P. (2013). Neutrophil recruitment and function in health and inflammation. Nat. Rev. Immunol..

[10] Tritto G., Bechlis Z., Stadlbauer V., Davies N., Francés R., Shah N., Mookerjee R.P., Such J., Jalan R. (2011). Evidence of neutrophil functional defect despite inflammation in stable cirrhosis. J. Hepatol..

[11] Wu L., Gao X., Guo Q., Li J., Yao J., Yan K., Xu Y., Jiang X., Ye D., Guo J. (2020). The role of neutrophils in innate immunity-driven nonalcoholic steatohepatitis: Lessons learned and future promise. Hepatol. Int..

[12] van der Windt D.J., Sud V., Zhang H., Varley P.R., Goswami J., Yazdani H.O., Tohme S., Loughran P., O’Doherty R.M., Minervini M.I. (2018). Neutrophil extracellular traps promote inflammation and development of hepatocellular carcinoma in nonalcoholic steatohepatitis. Hepatology.

[13] von Meijenfeldt F.A., Stravitz R.T., Zhang J., Adelmeijer J., Zen Y., Durkalski V., Lee W.M., Lisman T. (2022). Generation of neutrophil extracellular traps in patients with acute liver failure is associated with poor outcome. Hepatology.

[14] Sarin S.K., Choudhury A., Sharma M.K., Maiwall R., Al Mahtab M., Rahman S., Saigal S., Saraf N., Soin A.S., Devarbhavi H. (2019). Acute-on-chronic liver failure: Consensus recommendations of the Asian Pacific association for the study of the liver (APASL): An update. Hepatol. Int..

[15] DeLong E.R., DeLong D.M., Clarke-Pearson D.L. (1988). Comparing the areas under two or more correlated receiver operating characteristic curves: A nonparametric approach. Biometrics.

[16] Biggins S.W., Kim W.R., Terrault N.A., Saab S., Balan V., Schiano T., Benson J., Therneau T., Kremers W., Wiesner R. (2006). Evidence-based incorporation of serum sodium concentration into MELD. Gastroenterology.

[17] von Meijenfeldt F.A., Lisman T. (2022). Unravelling the Role of Neutrophil Extracellular Traps in Acute Liver Failure. Cell. Mol. Gastroenterol. Hepatol..

[18] He L., Cai Q., Liang X., Xin J., Shi D., Ren K., Li Y., Chen J., Sun S., Guo B. (2023). ETS2 alleviates acute-on-chronic liver failure by suppressing excessive inflammation. J. Med. Virol..

[19] Yang L.Y., Luo Q., Lu L., Zhu W.W., Sun H.T., Wei R., Lin Z.F., Wang X.Y., Wang C.Q., Lu M. (2020). Increased neutrophil extracellular traps promote metastasis potential of hepatocellular carcinoma via provoking tumorous inflammatory response. J. Hematol. Oncol..

[20] Hilscher M.B., Shah V.H. (2020). Neutrophil Extracellular Traps and Liver Disease. Semin. Liver Dis..

[21] Makkar K., Tomer S., Verma N., Rathi S., Arora S.K., Taneja S., Duseja A., Chawla Y.K., Dhiman R.K. (2020). Neutrophil dysfunction predicts 90-day survival in patients with acute on chronic liver failure: A longitudinal case-control study. JGH Open.

[22] Wu W., Sun S., Wang Y., Zhao R., Ren H., Li Z., Zhao H., Zhang Y., Sheng J., Chen Z. (2021). Circulating Neutrophil Dysfunction in HBV-Related Acute-on-Chronic Liver Failure. Front. Immunol..

[23] Vats R., Kaminski T.W., Brzoska T., Leech J.A., Tutuncuoglu E., Katoch O., Jonassaint J., Tejero J., Novelli E.M., Pradhan-Sundd T. (2022). Liver-to-lung microembolic NETs promote gasdermin D-dependent inflammatory lung injury in sickle cell disease. Blood.

[24] Klimiankou M., Skokowa J. (2021). Old drug revisited: Disulfiram, NETs, and sepsis. Blood.

[25] Zhang R., Sun C., Han Y., Huang L., Sheng H., Wang J., Zhang Y., Lai J., Yuan J., Chen X. (2023). Neutrophil autophagy and NETosis in COVID-19: Perspectives. Autophagy.

[26] Guiducci E., Lemberg C., Küng N., Schraner E., Theocharides A.P.A., LeibundGut-Landmann S. (2018). Candida albicans-Induced NETosis Is Independent of Peptidylarginine Deiminase 4. Front. Immunol..

[27] Thomson A.H. (1995). Human recombinant DNase in cystic fibrosis. J. R. Soc. Med..

[28] Davis J.C., Manzi S., Yarboro C., Rairie J., Mcinnes I., Averthelyi D., Sinicropi D., Hale V.G., Balow J., Austin H. (1999). Recombinant human Dnase I (rhDNase) in patients with lupus nephritis. Lupus.

[29] Wang C.Y., Lin T.T., Hu L., Xu C.J., Hu F., Wan L., Yang X., Wu X.F., Zhang X.T., Li Y. (2023). Neutrophil extracellular traps as a unique target in the treatment of chemotherapy-induced peripheral neuropathy. EBioMedicine.

[30] Ye D., Yao J., Du W., Chen C., Yang Y., Yan K., Li J., Xu Y., Zang S., Zhang Y. (2022). Neutrophil Extracellular Traps Mediate Acute Liver Failure in Regulation of miR-223/Neutrophil Elastase Signaling in Mice. Cell. Mol. Gastroenterol. Hepatol..

[31] Wang Y., Shi C., Guo J., Zhang D., Zhang Y., Zhang L., Gong Z. (2024). IDH1/MDH1 deacetylation promotes acute liver failure by regulating NETosis. Cell. Mol. Biol. Lett..

[32] Dwivedi D.J., Toltl L.J., Swystun L.L., Pogue J., Liaw K.L., Weitz J.I., Cook D.J., Fox-Robichaud A.E., Liaw P.C., Canadian Critical Care Translational Biology Group (2012). Prognostic utility and characterization of cell-free DNA in patients with severe sepsis. Crit. Care..

[33] Jiménez-Alcázar M., Kim N., Fuchs T.A. (2017). Circulating Extracellular DNA: Cause or Consequence of Thrombosis?. Semin. Thromb. Hemost..

[34] Hua X., Zhou H., Wu H.C., Furnari J., Kotidis C.P., Rabadan R., Genkinger J.M., Bruce J.N., Canoll P., Santella R.M. (2024). Tumor detection by analysis of both symmetric- and hemi-methylation of plasma cell-free DNA. Nat. Commun..

[35] Okkonen M., Lakkisto P., Korhonen A.M., Parviai-nen I., Reinikainen M., Varpula T., Pettilä V., FINNALI Study Group (2011). Plasma cell-free DNA in patients needing mechanical ventilation. Crit. Care..

[36] Tillack K., Naegele M., Haueis C., Schippling S., Wandinger K.P., Martin R., Sospedra M. (2013). Gender differences in circulating levels of neutrophil extracellular traps in serum of multiple sclerosis patients. J. Neuroimmunol..

